# Supplementary material for: Quantifying the effect of interpregnancy maternal weight and smoking status changes on childhood overweight and obesity in a UK population-based cohort
Source: PLoS One. 2024 Oct 7;19(10):e0311677. doi: 10.1371/journal.pone.0311677 (PMC11458013; doi:10.1371/journal.pone.0311677)
Supplement: Attachment — Submitted filename: Response-to-reviewers-effect-IPchanges _final.docx [file pone.0311677.s001.docx]

Journal requirements:

When submitting your revision, we need you to address these additional requirements.

1. Please ensure that your manuscript meets PLOS ONE's style requirements, including those for file naming. The PLOS ONE style templates can be found at

<https://journals.plos.org/plosone/s/file?id=wjVg/PLOSOne_formatting_sample_main_body.pdf> and

<https://journals.plos.org/plosone/s/file?id=ba62/PLOSOne_formatting_sample_title_authors_affiliations.pdf>

Thank you, we have updated the formatting to meet PLOS ONE’s requirements.

2. We noted in your submission details that a portion of your manuscript may have been presented or published elsewhere. [This work was presented at the virtual Lancet Public Health Science conference in November 2021 and the submitted abstract was published (https://doi.org/10.1016/S0140-6736(21)02627-1). This work originally formed part of the thesis for a Doctor of Philosophy degree undertaken by Elizabeth Taylor and funded by NIHR Southampton Biomedical Research Centre and University of Southampton Primary Care, Population Sciences and Medical Education PhD studentship. The thesis is under embargo until October 2024.] Please clarify whether this [conference proceeding or publication] was peer-reviewed and formally published. If this work was previously peer-reviewed and published, in the cover letter please provide the reason that this work does not constitute dual publication and should be included in the current manuscript.

The abstract was reviewed by the conference organising committee for decision making on acceptance of abstract to the conference programme. The short abstract was published in a supplement of the Lancet which is the usual practice for this conference’s accepted abstracts every year. This does not constitute dual publication as the conference abstract does not include the full introduction, methods, results and discussion included in the paper.

3. We note that you have indicated that there are restrictions to data sharing for this study. For studies involving human research participant data or other sensitive data, we encourage authors to share de-identified or anonymized data. However, when data cannot be publicly shared for ethical reasons, we allow authors to make their data sets available upon request. For information on unacceptable data access restrictions, please see <http://journals.plos.org/plosone/s/data-availability#loc-unacceptable-data-access-restrictions>.

Before we proceed with your manuscript, please address the following prompts:

a) If there are ethical or legal restrictions on sharing a de-identified data set, please explain them in detail (e.g., data contain potentially identifying or sensitive patient information, data are owned by a third-party organization, etc.) and who has imposed them (e.g., a Research Ethics Committee or Institutional Review Board, etc.). Please also provide contact information for a data access committee, ethics committee, or other institutional body to which data requests may be sent.

b) If there are no restrictions, please upload the minimal anonymized data set necessary to replicate your study findings to a stable, public repository and provide us with the relevant URLs, DOIs, or accession numbers. Please see <http://www.bmj.com/content/340/bmj.c181.long> for guidelines on how to de-identify and prepare clinical data for publication. For a list of recommended repositories, please see <https://journals.plos.org/plosone/s/recommended-repositories>. You also have the option of uploading the data as Supporting Information files, but we would recommend depositing data directly to a data repository if possible.

Please update your Data Availability statement in the submission form accordingly

We have edited the data availability statement as follows: The study’s ethical approval from the Faculty of Medicine Ethics Committee, University of Southampton and the Health Research Authority restricts public sharing of the raw data used in this study. To request access conditional on approval from the appropriate institutional ethics, research governance processes and data owners, please email [rgoinfo@soton.ac.uk](mailto:rgoinfo@soton.ac.uk).

4. Please review your reference list to ensure that it is complete and correct. If you have cited papers that have been retracted, please include the rationale for doing so in the manuscript text, or remove these references and replace them with relevant current references. Any changes to the reference list should be mentioned in the rebuttal letter that accompanies your revised manuscript. If you need to cite a retracted article, indicate the article’s retracted status in the References list and also include a citation and full reference for the retraction notice.
Thank you, we have reviewed the reference list as suggested.
[Note: HTML markup is below. Please do not edit.]

Reviewers' comments:
Reviewer #1:

Introduction: The introduction is clear and concise, but the literature cited is very global, none on the context where the study is conducted. Suggestion: include more contextual information including the problem and the gaps that the study addresses.
Thank you. Further detail has now been included in the introduction to address the reviewer’s comments (lines 50-54 and 58-63). These pick up on the specific prevalence of children with obesity in the UK and add more contextual detail.
Methods: Very well and clearly detailed. Thank you.
Results: Well detailed, aligned with the objectives and statistical analysis. However, quite difficult to follow, the author should add sub-topics representing the different sub-objectives / scenarios for clarity.
Thank you, sub-headings have now been added to aid clarity.

Reviewer #2:

An excellent paper. Well written, conceived and interesting. Results support the need for good pre- / inter-conception strategies to optimise maternal health and mitigate childhood risks. Thank you.
A few minor comments and suggests for the author(s):
Methods:
1. What is your rationale for excluding women who conceived via artificial reproductive therapy? This should be more clearly described and discussed in the paper. With increasing maternal age, more women are seeking to conceive via ART. Often BMI restrictions in the private sector are less closely adhered to than in the NHS. It is important that full representation of the pregnant population is described.

Thank you. Women who conceived one or both of their first two pregnancies using assisted reproductive technologies have been excluded to reduce any potential for residual confounding because of unmeasured health changes associated with this very heterogeneous group of interventions. Few exclusions were made for this reason (n = 309). The manuscript has been updated to be more specific for the reason for this exclusion (lines 100-102).

2. Exposure assessment - please state that the chosen BMI classes reflect a truncated WHO classification (REF).

Thank you, this has now been included as suggested (lines 117-118).

Results:
1. Table 3: gestational age could be more meaningful to present as weeks and days depending on readership. It may be clearer to include both this in additional to gestational age in days.
Thank you. For clarity, a footnote has been added to the Table to state that 280 days is equivalent to 40 weeks gestation.

2. Table 3: please check that units are clearly presented for all variables (e.g) weight change between pregnancies (%) in table 3.

Thank you, the second line of the table heading was omitted in error and has now been re-instated to show “All figures are proportions (%) unless stated otherwise.”

3. Abbreviations 3 and 4 are not described in the footnotes for table 3, please amend.

Thank you, these have now been replaced with a description, to be consistent with the entries for the second child. The abbreviations in the table have also been updated.

4. What was your rationale for significant weight gain (3 kg or more)? Please describe why in the manuscript.
Thank you. A brief description of the rationale with appropriate references has now been added to the manuscript (lines 291-293).

Discussion:
Well thought through and presented. Further discussion could include influence of recent maternity and public health strategies to support your proposed interventions. For example, the Women’s Health Strategy for England.
<https://www.gov.uk/government/publications/womens-health-hubs-information-and-guidance/womens-health-hubs-core-specification>

Thank you very much. A brief reference to this has now been included in the paper in the context of delivering interventions (lines 317-319).
